# Biliary Reconstruction with Hepaticoduodenostomy Versus Hepaticojejunostomy After Choledochal Cyst Resection: A Narrative Review

**DOI:** 10.3390/jcm13216556

**Published:** 2024-10-31

**Authors:** Nicholas Iglesias, Carlos Theodore Huerta, Royi Lynn, Eduardo A. Perez

**Affiliations:** Division of Pediatric Surgery, DeWitt Daughtry Family Department of Surgery, Miller School of Medicine, University of Miami, Miami, FL 33136, USActh62@miami.edu (C.T.H.);

**Keywords:** choledochal cyst, hepaticoduodenostomy, hepaticojejunostomy, pediatric, biliary reconstruction

## Abstract

Choledochal cysts (CCs), a congenital anomaly resulting in the abnormal dilation of the biliary ductal system, are most often identified in patients younger than 10 years of age. Regardless of clinical presentation, the cornerstone of therapy for CCs is complete surgical excision with reconstruction with either hepaticoduodenostomy or hepaticojejunostomy. Although both procedures are used by surgeons for the correction of CCs, evidence on clinical outcomes following both approaches is inconclusive as to which may offer superior reconstruction. This narrative review aims to compare the current literature regarding both approaches by evaluating their anatomic and operative considerations, as well as their perioperative, postoperative, and long-term outcomes. Future studies should closely focus on long-term, comparative outcomes, including the risk of biliary malignancy, and refine techniques to minimize complications, such as biliary reflux and bowel obstruction, in order to improve care for pediatric patients undergoing treatment for CCs.

## 1. Introduction

Occurring as a congenital anomaly resulting in the abnormal dilation of the biliary ductal system, choledochal cysts (CCs) are most often identified in patients younger than 10 years of age. These malformations are four times more common in females and have a higher prevalence in some populations, including patients of Eastern Asian descent [1]. Various etiologies are theorized to contribute to the development of CCs, including anomalous pancreaticobiliary union (APBDU), congenital bile duct stenosis, weak bile duct walls, and distal biliary obstruction [2,3]. Patients with CCs can either present with symptoms related to biliary stasis and reflux or may be identified incidentally through routine prenatal ultrasonography. 

Regardless of clinical presentation, the cornerstone of current therapy for CCs is complete surgical excision with reconstruction [4]. This treatment modality allows for risk mitigation from biliary stasis and malignant cyst transformation and provides symptomatic relief [3]. The two most common operative strategies for reconstruction, hepaticoduodenostomy and hepaticojejunostomy, are both undertaken for complete cyst excision and restoring biliary drainage. While both procedures are used by surgeons for the correction of CCs, evidence on clinical outcomes following both approaches is inconclusive as to which may offer superior reconstruction. Cyst excision with hepaticoduodenostomy is often cited as being technically less challenging, given the single anastomosis and more convenient postoperative endoscopic access to assess for strictures or stones [5]. Conversely, cyst excision with hepaticojejunostomy is historically the gold standard for biliary reconstruction and has a greater amount of associated older literature regarding its safety and efficacy.

This narrative review aims to compare the two approaches by evaluating their anatomic and operative considerations, as well as their perioperative, postoperative, and long-term outcomes. 

## 2. Methods

Manuscripts published between 1978 and 22 February 2024 were identified in the National Institute of Health National Library of Medicine PubMed and MEDLINE databases. Search terminology included (“hepaticojejunostomy” OR “hepaticoduodenostomy”) AND “choledochal cyst” to maximize search sensitivity. Manuscripts met inclusion criteria if they described surgical correction for patients less than 18 years old with choledochal cysts, were published in English or had a full, translated manuscript available online, and analyzed a human study population. Manuscripts were excluded if they described surgical correction of another biliary pathology or alternative surgical/procedural correction of choledochal cysts (e.g., endoscopic drainage or choledochojejunostomy/cystojejunostomy), were not available in full in English, were individual case reports or descriptive reviews, did not separate hepaticoduodenostomy and hepaticojejunostomy outcomes when reporting data, or analyzed an animal model.

## 3. Discussion

### 3.1. Anatomic and Preoperative Considerations

The pathogenesis of choledochal cysts is not currently well defined, although multiple hypotheses exist [6]. Anomalous pancreaticobiliary mal-junction and congenital bile duct stenosis are the two prevailing hypotheses for mechanical causes of choledochal cysts. Pancreaticobiliary mal-junction is thought to result in an abnormally long common channel, which is at increased risk of pancreatic and biliary reflux. In 1969, Babbitt et al. were the first to comment on the inflammatory response of a long common channel to prolonged pancreatic enzyme exposure, ultimately leading to biliary ductal degeneration and dilatation [6,7,8]. Prior studies suggest that only 50–80% of choledochal cysts are associated with pancreaticobiliary mal-junction [9]. Alternatively, congenital or acquired (in the adult population) stenosis of the bile ducts has been proposed to cause proximal biliary ductal dilation and cyst formation [7,9,10]. 

Current data suggest that a genetic component may be related to the congenital development of choledochal cysts, given the increased incidence of choledochal cysts in Eastern Asia as compared to Western countries. Moreover, other reports have suggested a familial incidence of congenital biliary pathology in Japan [6,9,11,12]. One previous study reviewed numerous genetic studies that identified multiple chromosomal anomalies (e.g., genes HNF1B, EHBP1, and APC) and transcriptomic signatures (e.g., ERBB2 and WNT11 hub genes) associated with choledochal cysts [6].

Choledochal cysts may be identified at any age, from fetus to adulthood. Prenatal diagnoses are often discovered incidentally during routine anatomic ultrasound (US) exams and prompt neonatal imaging with repeat US or magnetic resonance cholangiopancreatography (MRCP) [11]. Neonatal presentation often mimics biliary atresia with sequelae, including obstructive jaundice, acholic stools, and/or hepatomegaly [11,13]. Classically, although present in as few as 15% of cases, choledochal cysts may present in children with a triad of abdominal pain, jaundice, and a palpable abdominal mass [11,14]. More than 80% of choledochal cysts are identified in children < 10 years old [15]. Children are more likely to have two of the three features of the classic triad than adults (82% vs 25%) [16]. Fever, nausea, and vomiting may also be present in children and adults due to concurrent ascending cholangitis or pancreatitis [11,14,16]. Spontaneous rupture and subsequent peritonitis have been described in 1–12% of patients and often confounds US findings due to biliary decompression [14,17]. 

Symptomatology, as described above, or concerning pre-natal imaging findings prompts ultrasonography, although sensitivity varies from 71–97% [14]. Precise pancreatico-biliary ductal anatomic imaging is recommended if diagnosis is confirmed by US or if the US is negative but a diagnosis of choledochal cysts is still strongly suspected. MRCP has largely predominated as the imaging modality of choice, although endoscopic retrograde cholangiopancreatography (ERCP), computed tomography cholangiopancreatography (CTCP), and endoscopic ultrasound (EUS) have also been described in other reports [7,11,14,18,19].

In 1959, the first classification of choledochal cysts was described by Alonso-Lej et al. [20]. In 1977, Todani et al. expanded upon Alonso-Lej et al.’s classification system, expanding from three to five types of choledochal cysts [21]. The Todani classification, which now serves as the most-used choledochal cyst classification, is described as outlined in Table 1.

### 3.2. Operative Considerations

Choledochal cysts diagnosed in the prenatal period are often managed once the patient has surpassed 5 kg or 3 months of age. Concern for biliary atresia or biliary obstruction will prompt more urgent surgical intervention [11]. Those with concomitant cholangitis and/or pancreatitis require medical management of concurrent cholangitis and/or pancreatitis and prompt surgical intervention. Asymptomatic patients with imaging-confirmed choledochal cysts may undergo surgical management on an elective basis, but close follow-up is required. It is recommended that all choledochal cysts undergo surgical intervention, due to a >10% lifetime risk of malignancy [22]. Management of choledochal cysts by experienced surgical teams and hospitals with multidisciplinary care is recommended for effective perioperative management, which may necessitate additional procedures by gastroenterology or interventional radiology.

Surgical management of Type I choledochal cysts has been the subject of significant debate. Biliary reconstruction may be approached in several ways, including cystoenterostomy or hepaticoenterostomy. Cystoenterostomy was thought to provide sufficient biliary drainage, but ultimately, patients remained at an unacceptably elevated risk of malignancy when compared to complete cyst excision. Ten Hove et al.’s meta-analysis of malignancy in choledochal malformations found that patients who underwent choledochoenterostomy suffered a nearly four-times increased malignancy risk, compared to those who underwent cyst excision and biliary reconstruction (odds ratio 3.97, *p* = 0.006) [22]. Patients who have previously undergone cystoenterostomy are advised to undergo reoperation for completion cystectomy and biliary reconstruction to reduce the lifetime risk of a biliary malignancy. Complete cyst excision is imperative to reduce the overall lifetime malignancy risk; therefore, distal common bile duct margins are often within the pancreas itself, near the junction of the pancreatic duct [11,13]. Once the cyst is mobilized from the portal vein and hepatic artery up to the level of the liver, the proximal duct is transected, and the gallbladder is also removed. The creation of a wide stoma as high as the hepatic duct confluence for enteric anastomosis has been advocated for by many surgeons [13,23,24,25].

**Figure 1:**
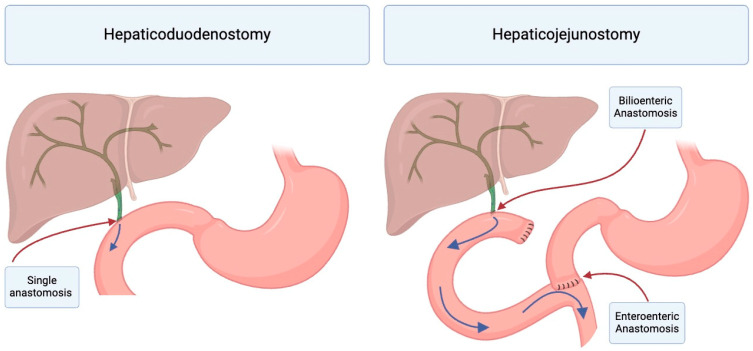
Biliary Reconstruction Methods after Choledochal Cyst Resection. Once the cyst is mobilized from the portal vein and hepatic artery up to the level of the liver, the proximal duct is transected, and the gallbladder is also removed. After resection of the choledochal cyst and cholecystectomy, biliary reconstruction is achieved via either hepaticoduodenostomy or hepaticojejunostomy. Hepaticoduodenostomy involves a single anastomosis from the common bile duct to the duodenum. Conversely, hepaticojejunostomy includes the transection of the jejunum distally brought up as a limb anastomosed to the common bile duct (bilioenteric) and a distal enteroenteric anastomosis for alimentary function.

Hepaticoenterostomy may be subdivided into hepaticoduodenostomy (with or without jejunal interposition) and Roux-en-Y hepaticojejunostomy (Figure 1). Hepaticoduodenostomy was initially advocated for by Dr. Todani in 1981, due to its “capability of preventing cholangitis”, resulting in a “better physiological state” and “fewer post operative complications” [24]. Hepaticoduodenostomy is a common approach to biliary reconstruction and is associated with technical advantages, including reduced operative time, fewer number of anastomoses, and feasibility of future endoscopic procedures [11,15]. The position of hepaticoduodenostomy relative to the pylorus is not standardized at this time, with some high-volume centers positioning it 2 cm distal to the pylorus and others 3 cm distal to the pylorus [26]. Concerns for the reflux of duodenal contents into the biliary tract, cholangitis, and mucosal inflammation subsequently prompted the consideration of jejunal interposition and hepaticojejunostomy. Hepaticoduodenostomy with jejunal interposition was hypothesized to provide the benefits of a hepaticoduodenostomy while simultaneously avoiding the malabsorption of hepaticojejunostomy and reflux associated with hepaticoduodenostomy. However, early attempts demonstrated patients experiencing excessive bile reflux gastritis [5,27]. A valved jejunal interposition hepaticoduodenostomy has been described, although it is not commonly used at this time [28]. Hepaticojejunostomy is thought to avoid chronic biliary tract inflammation and reduce the risk of cholangitis. Ultimately, Todani et al. in 2002 reported a single patient who developed biliary cancer 19 years following choledochal cyst excision and hepaticoduodenostomy, suspecting its underlying etiology to be inflammation secondary to the reflux of duodenal contents and activated pancreatic enzymes into the intrahepatic bile ducts. This subsequently prompted him to transition to performing hepaticojejunostomy for subsequent choledochal cysts [29].

Traditionally, choledochal cysts were managed with open surgery, but the widespread adoption of minimally invasive surgery has prompted surgeons to perform choledochal cyst excision and biliary reconstruction via laparoscopy and robotic surgery. One previous study reported that laparoscopic surgery offered a shorter hospital length of stay, lower total cost, and reduced complications [15]. Hepaticoduodenostomy is the most common method of biliary reconstruction when a minimally invasive approach is utilized, and hepaticojejunostomy is the most common method of biliary reconstruction during an open approach [15]. On a national scale, laparoscopic surgery is associated with reductions in hospital length of stay, cost, need for additional procedures, sepsis, and respiratory disorders for these reconstructive surgeries [15].

Todani Type II choledochal cysts may be treated with isolated cyst resection, although additional biliary resection is required in up to 58% of cases, and biliary reconstruction may be required [30]. Type III choledochal cysts, choledochocele, have a reduced risk of malignant transformation, compared to other types of choledochal cysts. Endoscopic management with sphincterotomy of the common bile duct with the de-roofing of the cyst or sphincterotomy alone is now the standard of care for choledochocele [31,32]. Todani Type IV requires complete cyst excision and possibly partial hepatectomy as directed by preoperative imaging demonstrating the location of any intrahepatic lesions. Biliary reconstruction may be recommended for extrahepatic Todani Type IV lesions. Localized Todani Type V may also be treated with partial hepatectomy with success [33,34]. Diffuse Type V is not amenable to surgical resection and biliary reconstruction and ultimately requires liver transplantation for a cure [33]. The management of Todani Types I–V choledochal cysts are summarized in Table 2.

### 3.3. Postoperative Outcomes of Hepaticoduodenostomy and Hepaticojejunostomy

As of the current year, the two primary methods of biliary reconstruction for choledochal cyst are hepaticoduodenostomy and hepaticojejunostomy. For decades, there has been significant debate on the superior operative technique, prompting numerous investigations on short- and long-term outcome differences between the two procedures. Perioperative measures that are compared between the two procedures include operative time, blood loss, hospital length of stay, and time to diet initiation. Long-term outcomes compared between the two procedures include bile reflux gastritis, cholangitis, leak, anastomotic stricture, reoperation, bowel obstruction, and development of biliary neoplasms. Table 3 summarizes outcome differences between hepaticoduodenostomy and hepaticojejunostomy.

#### 3.3.1. Perioperative Outcomes

The operative time of hepaticoduodenostomy is almost universally reduced when compared to hepaticojejunostomy, largely due to the reduced number of anastomoses (1 vs. 2). Meta-analyses, along with multiple high-volume center studies, report statistically significant differences in operative times, with between 55 and 180 min of reductions when conducting hepaticoduodenostomy, compared to hepaticojejunostomy [26,35,36,37,38,39,40,41,42,43]. Meta-analyses cite the mean operative time for hepaticoduodenostomy to be from 95–336 min, compared to 212–390 min for hepaticojejunostomy [35,36]. Conversion of the operative technique from open to laparoscopic is associated with a significant learning curve, with one high-volume center reporting the operative time decreasing from 422 to 282 min after multiple years of laparoscopic practice [38].

Potentially secondary to the reduced operative time or the feasibility of a laparoscopic approach, hepaticoduodenostomy is also associated with an 11–49 mL reduced intraoperative blood loss. Mean operative bleeding during hepaticoduodenostomy ranges from 10–26 mL, compared to 21–307 mL during hepaticojejunostomy [35,38]. However, there are multiple other meta-analyses and studies from high-volume centers that report no significant difference in intraoperative blood loss between hepaticoduodenostomy and hepaticojejunostomy [5,36,37,39,44].

Recent meta-analyses, alongside other studies, report no statistically significant delay in the return of the diet [35,36,37,38,44]. In 2015, one group reported an intentional delay of oral feeding due to limited experience with hepaticoduodenostomy, although a subsequent study by the same team later reported no delay in the return of the diet [38,39].

Total hospital length of stay is reportedly reduced in patients that underwent hepaticoduodenostomy. Meta-analyses report anywhere from a 0.3-day to 2.18-day reduction in total hospital length of stay in patients undergoing hepaticoduodenostomy, compared to hepaticojejunostomy [5,35,36]. This difference in length of stay was not identified in single-center studies, nor in a retrospective dataset analysis [15,26,37,39].

#### 3.3.2. Long-Term Outcomes

The most-cited complications of hepaticoduodenostomy are biliary reflux and gastritis, due to the proximity of the anastomosis to the pylorus. Meta-analyses, as well as studies from multiple high-volume centers, reported an increased incidence of biliary reflux in patients who underwent hepaticoduodenostomy, with an odds ratio ranging between 6.24 and 19.14 [5,26,27,35,36,38,40,41,45,46,47]. Incidence of biliary reflux was cited to be between 3.8% and 100% of patients who underwent hepaticoduodenostomy. In one prior study, patients after hepaticoduodenostomy were endoscopically evaluated for duodenogastric regurgitation (DGR), and it was found that 17/17 patients had DGR, with 14/17 being symptomatic [46]. This complication is persistent in hepaticoduodenostomy, even if a jejunal interposition is utilized [27]. There are proponents of extensive kocherization and placing the anastomosis more distal to the pylorus to reduce the risk of reflux and gastritis [26]. Close endoscopic follow-up for the monitoring and management of biliary reflux is recommended in patients with hepaticoduodenostomy.

In 1981 Todani et al. originally reported hepaticoduodenostomy, especially with a wide stoma, to be associated with an equivalent rate of cholangitis, compared to hepaticojejunostomy [24]. This hypothesis has withstood the test of time, with meta-analyses, as well as studies from multiple high-volume centers, reporting no statistically significant difference in the rate of cholangitis between hepaticoduodenostomy and hepaticojejunostomy [5,15,24,26,35,36,37,38,39,40,41,42,48,49]. Some groups advocate for allowing the common hepatic duct diameter to dictate the approach to biliary reconstruction, with a cutoff of 10 mm being the upper limit for hepaticoduodenostomy. They hypothesize that a larger diameter would facilitate an easier reflux of duodenal contents into the common hepatic duct, therefore causing an increased risk of cholangitis [45].

The presence of a Roux limb and the known risk of intestinal obstruction in other patients undergoing Roux-en-Y purports the hypothesis that hepaticojejunostomy may confer an increased risk of internal hernia or intestinal obstruction. One meta-analysis reported that postoperative intestinal obstruction was reported to be more common in patients with hepaticojejunostomy, compared to hepaticoduodenostomy (5.5% vs. 0%) [36]. Previous meta-analyses reported intestinal obstruction in 5.1–7.7% of patients with hepaticojejunostomy and 0% in hepaticoduodenostomy. However, two studies did not elucidate a statistically significant difference in bowel obstruction rates following hepaticoduodenostomy and hepaticojejunostomy [5,35].

The risk of postoperative biliary leak has not been found to be significantly different between hepaticoduodenostomy and hepaticojejunostomy [5,26,35,36,37,38,39,41]. The risk of anastomotic stricture has not been found to be significantly different between hepaticoduodenostomy and hepaticojejunostomy [5,36,37,38,48,49,50]. Santore et al. reported that hepaticojejunostomy was associated with increased reoperation [37]. However, subsequent meta-analyses and studies from high-volume centers have not found a significant difference in the rate of reoperation between hepaticoduodenostomy and hepaticojejunostomy [5,35,36,38,39].

The most feared complication of hepaticoduodenostomy is the subsequent development of biliary neoplasms. The hypothesis underpinning this concern is that hepaticoduodenostomy subjects the intrahepatic bile ducts to chronic inflammation due to chronic exposure to duodenal contents and activated pancreatic enzymes. Todani et al., the original supporters of hepaticoduodenostomy, disavowed this method of biliary reconstruction in 2002 after a patient developed biliary cancer 19 years following choledochal cyst excision and hepaticoduodenostomy [29]. Several high-volume centers with prolonged patient follow-up following hepaticoduodenostomy and hepaticojejunostomy (multiple following patients for decades) have not reported biliary cancer following reconstruction [25,36,38,45,48,51]. Furthermore, hepaticoduodenostomy does not appear to be the only method of biliary reconstruction associated with biliary neoplasms. In 1999, one study analyzed 23 patients who developed biliary cancer 1–19 years following partial or complete choledochal cyst excision with or without biliary reconstruction. Of the 23 patients who developed biliary cancer following partial or complete choledochal cyst excision with or without biliary reconstruction, only 2 underwent hepaticoduodenostomy, whereas 13 underwent hepaticojejunostomy. Biliary cancer at the hepatic hilum or anastomotic site was found in six patients with hepaticojejunostomy and in only one with hepaticoduodenostomy [52]. These data suggest that the risk of biliary malignancy following hepaticoduodenostomy may not be solely due to the biliary reconstruction itself. Some authors propose that patients with choledochal cysts may have a field risk of biliary neoplasms that is independent of the approach to biliary reconstruction [37]. Long-term endoscopic follow-up of patients following choledochal cyst excision is supported, not only for the evaluation of biliary reflux but for biliary malignancy screening as well [36,51].

## 4. Conclusions

Ultimately, surgical excision and biliary reconstruction remain the cornerstones of treatment for pediatric patients with choledochal cysts. While reconstruction via hepaticoduodenostomy and hepaticojejunostomy both aim to restore biliary outflow, further data are needed to better characterize the selection of each approach relevant to their known advantages and impact on clinical outcomes. Hepaticoduodenostomy is generally associated with shorter operative times, reduced blood loss, and potentially shorter hospital stays, compared to hepaticojejunostomy. However, these benefits, in terms of reduced operative time and hospital stay, are not universally consistent across all studies. While hepaticoduodenostomy retains advantages, such as relative technical ease and feasibility for laparoscopic approaches, it does not appear to confer significant immediate benefits with regards to time to diet resumption or overall recovery time when compared to hepaticojejunostomy. Furthermore, the incidence of biliary reflux and gastritis due to the proximity of the anastomosis to the pylorus appears to be higher in patients treated with hepaticoduodenostomy. This reflux may contribute to chronic inflammation, although the risk of biliary malignancy in this context remains debatable. Conversely, hepaticojejunostomy may be associated with a higher risk of bowel obstruction but lower incidences of biliary reflux and cholangitis in most reports. Further high-quality, multicenter, randomized controlled trials are needed to provide clearer guidance on the optimal anatomic and patient selection criteria for each surgical approach in the context of available institutional resources. As newer technologies and approaches, such as robotic-assisted techniques, become more widely disseminated, studies into the applicability and efficacy of such technologies in the pediatric population for choledochal cyst resection should be investigated. Moreover, future studies should closely focus on long-term, comparative outcomes, including the risk of biliary malignancy; refine techniques to minimize complications, such as biliary reflux and bowel obstruction; and improve care for pediatric patients undergoing treatment for choledochal cysts.

## Figures and Tables

**Table 1:** Todani choledochal cyst classification.

Todani Cyst Classification:	Description
Type Ia	Saccular dilation of the common bile duct
Type Ib	Segmental choledochal dilation
Type Ic	Diffuse/cylindrical dilation of the majority of extra-hepatic ducts
Type II	Diverticulum of the extra-hepatic duct
Type III	Choledochocele of the duodenal portion of the CBD
Type IVa	Multiple intra- and extra-hepatic ductal cysts
Type IVb	Multiple extra-hepatic ductal cysts
Type V	Intrahepatic bile duct cyst(s). If multiple, termed Caroli’s disease

Abbreviations: CBD, common bile duct.

**Table 2:** Management of choledochal cysts according to the Todani classification.

Todani Cyst Classification:	Management
Type I	Cyst resection and biliary reconstruction
Type II	Isolated cyst resection ± biliary reconstruction
Type III	Endoscopic (sphincterotomy ± cyst de-roofing)
Type IVa	Cyst resection, partial hepatectomy, and biliary reconstruction
Type IVb	Cyst resection and biliary reconstruction
Type V (localized)	Partial hepatectomy (if anatomy amenable)
Type V (diffuse)	Liver transplantation

**Table 3:** Perioperative and long-term outcome comparisons between hepaticoduodenostomy and hepaticojejunostomy.

Perioperative Outcome	Hepaticoduodenostomy	Hepaticojejunostomy	References
Operative Time	Reduced operative time (55–180-min reduction)	Longer operative time	[26,35,36,37,38,39,40,41,42,43] [38] also reports a learning curve of the laparoscopic approach
Blood Loss	No difference		[5,35,36,37,38,39,44]
Hospital Length of Stay	Decreased hospital LOS (0.3–2.18)	Longer hospital LOS	Meta-analyses that identify a significant difference [5,35,36] Single-center/retrospective studies that do not identify a significant difference [15,26,37,39]
Time to Diet Initiation	No difference	No difference	[35,36,37,38,44]
Long-Term Outcome	-	-	
Bile Reflux Gastritis	Increased risk (OR 6.24–19.14, incidence between 3.8% and 100%)	Minimal risk	[5,26,27,35,36,38,40,41,45,46,47]
Risk of Cholangitis	No difference		[5,15,24,26,35,36,37,38,39,40,41,42,48,49]
Bile Leak Incidence	No difference		[5,26,35,36,37,38,39,41]
Anastomotic Stricture Rate	No difference		[5,36,37,38,48,49,50]
Bowel Obstruction	Minimal risk of bowel obstruction; not statistically significant	Potential increased risk of bowel obstruction; not statistically significant	[5,35]
Need for Reoperation	No difference		[5,35,36,38,39]
Development of Biliary Neoplasms	Hypothetical risk of biliary neoplasm, although not statistically confirmed or reproduced. Patients remain at a hypothesized lifetime risk.	Possible decreased risk of biliary neoplasm, although not statistically confirmed. Patients remain at a theorized lifetime risk due to a theorized field defect	[25,36,37,38,45,48,51,52]

Abbreviations: odds ratio, OR; length of stay, LOS.
